# Assessment of Heavy Metal Contamination, Distribution, and Source Identification in Surface Sediments from the Mid–Upper Reaches of the Yellow River

**DOI:** 10.3390/toxics13030150

**Published:** 2025-02-23

**Authors:** Junzhang Wang, Ling Tao, Hanru Ren, Xiangyu Xue, Zhijie Yang, Yucheng Jiang, Jun Ren

**Affiliations:** 1 School of Environmental and Municipal Engineering, Lanzhou Jiaotong University, Lanzhou 730070, China; wangjz@mail.lzjtu.cn (J.W.); taoling@mail.lzjtu.cn (L.T.); renhanru@163.com (H.R.); 11220143@stu.lzjtu.edu.cn (X.X.); 11220065@stu.lzjtu.edu.cn (Z.Y.); jiangyc6010@162.com (Y.J.); 2 Key Laboratory of Yellow River Water Environment in Gansu Province, Lanzhou Jiaotong University, Lanzhou 730070, China; 3 Gansu Hanxing Environmental Protection Co., Ltd., Lanzhou 730070, China

**Keywords:** Yellow River, sediments, heavy metal, spatial distribution, source analysis

## Abstract

River sediments serve as both a source and a reservoir for potential heavy metal pollutants, providing critical insights into the health of aquatic ecosystems. Heavy metal contamination in global river systems poses significant risks to human health through the food chain. This study investigates the contents of heavy metals, including Fe, Mn, Cu, Ni, Zn, Cr, Pb, and Cd, in surface sediment samples collected from the mid–upper reaches of the Yellow River, which flows through the provinces of Qinghai, Gansu, Ningxia, Inner Mongolia, Shaanxi, and Henan. We analyzed the contents and spatial distribution characteristics of these metals and assessed their pollution levels by identifying potential sources of contamination. The findings reveal elevated concentrations of Cr and Cd in the mid–upper reaches of the Yellow River, with particularly severe pollution observed in certain urban areas. The sources of these heavy metals are primarily linked to human activities related to production and daily life. This study offers valuable guidance for local pollution control and prevention strategies.

## 1. Introduction

Rivers have many functions in the water environment, such as ecological service, geochemical cycling, and providing habitats for animals and plants [1,2]. However, with rapid economic development and urban expansion in many developing countries, a large number of uncontrollable pollutants are artificially flowing into rivers, resulting in considerable challenges to the aquatic environment [3,4,5]. At present, heavy metals in river sediments are regarded as the key source of river pollutants. In view of the cumulative characteristics of pollutants in river sediments, pollutant levels can be monitored and evaluated by specific indicators [6,7,8]. Heavy metals in river sediments originate from both natural and anthropogenic sources, and given their persistent and far-reaching impacts on the environment, they have captured significant attention both domestically and internationally. These substances pose a long-term threat to the ecological balance of river systems and overall environmental quality, making them a focal point of research and environmental monitoring efforts around the world [9,10,11].

Heavy metals will quickly migrate from the water to the sediments and be adsorbed on the surfaces of particles, and then they further migrate and release into the water body through changes in the external environment, threatening the water ecosystem [12,13]. Therefore, river sediment can be regarded as the repository of heavy metals, which can be taken as the main research object for monitoring heavy metal pollutants in water ecosystems. Its spatial distribution characteristics, contents, and pollution levels will help to determine the source of heavy metals and play a key role in evaluating river pollution and potential ecological risks [14,15,16], and evaluating heavy metal pollution will also provide some guidance for water environment risk management [17,18].

The Yellow River is the second largest river in China, located at 96~19 east longitude and 32~42 north latitude. It originates in Qinghai Province, flows through Gansu, Ningxia, Inner Mongolia, Shanxi, Shaanxi, Henan, and Shandong provinces, and finally flows into the Bohai Sea [2]. As the most important river in northern China, the Yellow River provides water for 15% of the cultivated land and for nearly 160 million people in China [19]. Due to agricultural, urban, and industrial activities, the Yellow River has become polluted in the past decades, especially with heavy metal pollution [20,21]. Previously, some researchers analyzed heavy metal pollution in the sediments of the Yellow River, but the investigation was limited to only specific or typical small areas, such as estuaries, wetlands, nature reserves, etc. [22,23].

The sediment samples collected also came from different locations (the riverbed, the flood plain or alluvial area, or the main stream or tributaries) [24,25,26]. Therefore, a comprehensive study on the distribution characteristics and pollution levels of heavy metals in surface sediments from the mid–upper reaches of the Yellow River is of great significance to further reveal the overall pollution status of the Yellow River [27,28].

The main research purposes of this paper are as follows: (1) ascertain the regular pattern of heavy metal distribution along rivers by analyzing the spatial distribution characteristics of heavy metals in sediments; (2) reveal the enrichment characteristics of Cu, Fe, Mn, Ni, Zn, Cr, Pb, and Cd in the mid–upper reaches of the Yellow River; (3) clarify the toxicity risk grade of heavy metals in surface sediments from the mid–upper reaches of the Yellow River; and (4) judge the source of heavy metal pollution by multivariate statistical analysis.

## 2. Material and Methods

### 2.1. Study Area

**Figure 1:**
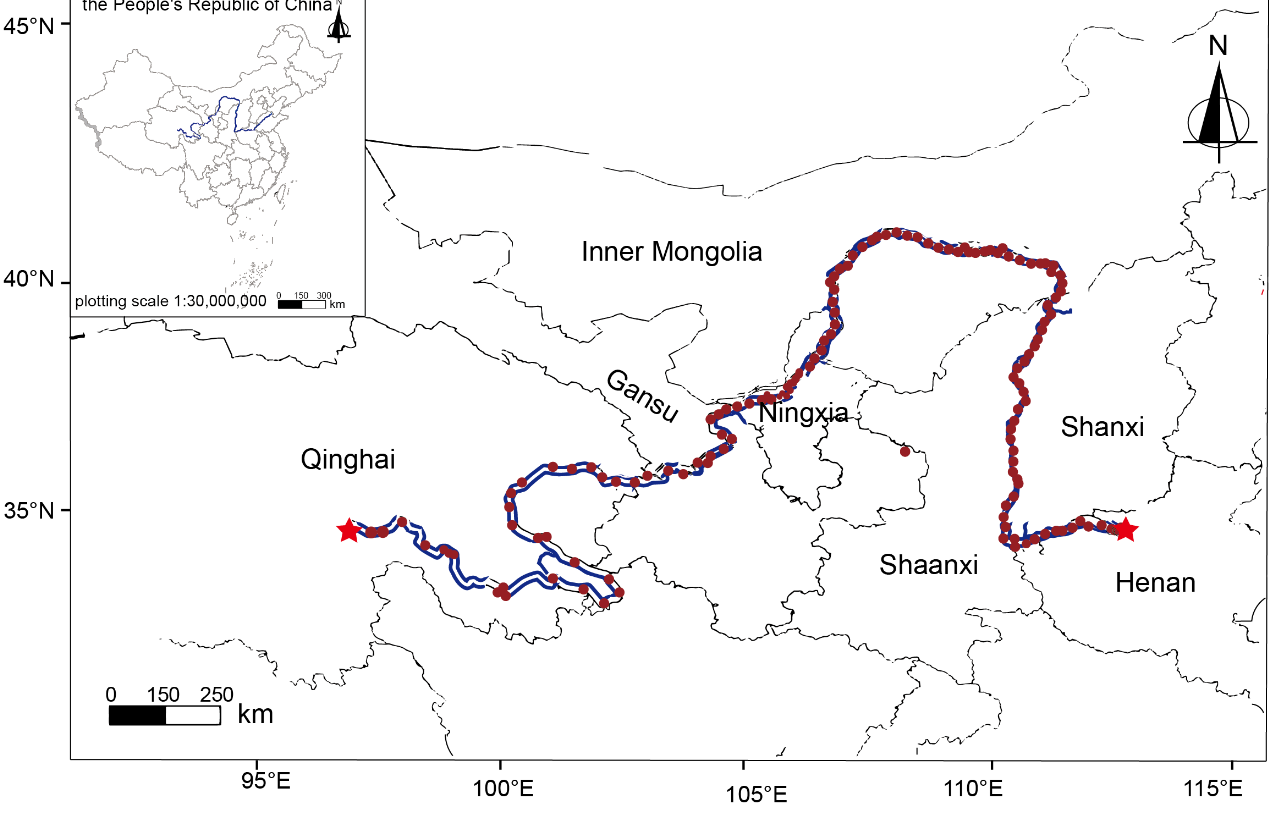
Study area and location of sampling sites. The map of China and local maps within China were generated by using the China map, sf, and ggplot2 software packages of R (with version 4.4.1, available at https://www.r-project.org/, accessed on 10 August 2024). The map of China in the resulting picture was sourced from the website of Chinese National Geography (website: http://www.dili360.com/, accessed on 20 December 2023). Red stars represent the starting and ending points of this study, and the circles represent the sampling points.

The mid–upper reaches of the Yellow River start from the source of the Yellow River in Bayan Har, Qinghai Province, pass through the seven provinces of Qinghai, Gansu, Ningxia, Inner Mongolia, Shaanxi, Shanxi, and Henan, and end in Mengjin District, Luoyang City, Henan Province, with a total length of approximately 4678 km and a drainage area of 772,000 square kilometers [29,30] (Figure 1). In recent years, the domestic sewage and industrial wastewater discharged into the mid–upper reaches of the Yellow River have increased year by year, and the water quality has deteriorated obviously. In addition, in order to provide protection for hydropower, flood control, and the water supply, a series of Yellow River dams have also changed the quantity and quality of river sediments [31,32]. In this study, the pollution situation, distribution characteristics, and sources of heavy metals in river sediments from the mid–upper reaches of the Yellow River were analyzed.

### 2.2. Collection of Samples

A total of 230 sampling points were evenly set up along the mid–upper reaches of the Yellow River. The setting was based on the principle of accessibility and in combination with administrative units. At the established sampling points, 226 sediment samples (0–5 cm) were collected from the riverbed using a grab gravity mud collector. The sediment samples were collected as mixed samples. Specifically, sediment samples were collected three times at different positions in the same section and then thoroughly mixed. Soil samples were also collected as mixed samples for control purposes. That is, three surface soil samples (2–10 cm) were collected at different locations in the same area and fully mixed. The sampling point number, sampling time, location, latitude and longitude, altitude, the landform unit where the sampling point is located, river width, water depth, and the distance between the sampling point and the shore were recorded in detail. Photos were taken of the river section where the sampling point was located and the surrounding landform (including vegetation status and erosion status). The collected sediment samples were packed in plastic bottles, and the soil samples were packed in polyethylene plastic bags. Information such as the sample number, sampling location, and date was marked on the plastic bottles or plastic bags. The samples were then taken back to the laboratory for air drying, grinding, and screening before proceeding with the next experimental analysis (see Supplementary Materials).

### 2.3. Sample Digestion

First, 0.5 g (to the nearest 0.0001) of the sample was precisely weighed using an electronic balance and then digested in a fluoroplastic crucible. The digestion steps are as follows: firstly, the soil sample was wetted with three drops of distilled water. Then, 10 mL of concentrated hydrochloric acid was added. The temperature was controlled at 90 °C on an electric heating plate, and the sample was heated to a viscous state at a constant temperature. Secondly, 10 mL of concentrated nitric acid (superior grade pure) was added, and the mixture was continuously heated until it became viscous. Next, 10 mL of hydrofluoric acid (grade pure) was added again, and the heating was continued until it was viscous. Finally, 10 mL of perchloric acid (grade pure) was added and heated until white smoke was exhausted. The digested sample is white or yellow, and it is sticky when the crucible is tilted. The digested sample was rinsed with water and poured into a funnel for filtration. The inner wall of the crucible was rinsed twice and the solution was poured into the funnel. When the solution in the funnel was less than one third, the filter paper with was rinsed water and this was repeated twice. Finally, the filter paper was taken out and the funnel was washed with distilled water, then distilled water was used to make a constant volume of 100 mL. It was shaken well and tested. Digestion was repeated 4 times.

### 2.4. Sample Determination

The contents of heavy metals such as Fe, Mn, Cu, Zn, Ni, Pb, Cd, and Cr in all the treated sediment and soil samples were determined by an inductively coupled plasma mass spectrometer (ICP-MS, Thermo Fisher Scientific, Waltham, MA, USA). All the samples were processed three times, the final data were their mean values, and the relative standard deviations were all within 10%.

### 2.5. Assessment Methods

There are many evaluation methods for heavy metal pollution in surface sediments, and the evaluation system is relatively perfect [22]. In this study, the most widely used enrichment factor method, geoaccumulation index method, potential ecological risk index method, and sediment quality guideline values were selected to evaluate the heavy metal pollution in sediments.

#### 2.5.1. Enrichment Factor Method (*EF*)

*EF* is an important and widely used method to evaluate the pollution degree of river sediment [33]. At present, the commonly used reference elements are Al, Fe, Mn, Mg, Ca, etc. In order to identify abnormal metal content, a normalized metal is used, and the Mn element is used as the reference material in this study [34]. The calculation method is as follows:

(1) $$EF=\frac{{\left(\frac{M}{Mn}\right)}_{sample}}{({\frac{M}{Mn})}_{background}}$$

where ${\left(\frac{M}{Mn}\right)}_{sample}$ is the ratio of measured values of a metal element M and Mn in the same sample, world average content of heavy metals in shale (Fe: 47,200 mg/kg, Mn: 850 mg/kg, Cu: 45 mg/kg, Ni: 68 mg/kg, Zn: 95 mg/kg, Cr: 90 mg/kg, Pb: 20 mg/kg, Cd: 0.30 mg/kg) was taken as the background value in this study, and ${\left(\frac{M}{Mn}\right)}_{background}$ is the ratio of background values of a metal element M and Mn. The evaluation criteria of the enrichment coefficient can be expressed in seven grades (Table 1) [35].

#### 2.5.2. Geoaccumulation Index Method (*I_geo_*)

The geological accumulation index (*I_geo_*) method was first proposed by Müller (1969) to evaluate the pollution degree of heavy metals in water sediments [36]. Its application formula is as follows:

(2) $$I_{geo}={log}_{2}\left(\frac{C_{n}}{{1.5B}_{n}}\right)$$

where *C_n_* is the measured content of heavy metal *n* and *B_n_* represents the geochemical background value of heavy metal *n*. In this study, the average content of heavy metals in the upper crust (Fe: 35,000 mg/kg, Mn: 600 mg/kg, Cu: 25 mg/kg, Ni: 20 mg/kg, Zn: 71 mg/kg, Cr: 35 mg/kg, Pb: 20 mg/kg, Cd: 0.10 mg/kg) was used as the background value [35]. Here, 1.5 is a correction index to consider the influence of different rocks on background values [37]. The *I_geo_* can be divided into seven grades to indicate the change in pollution degree (Table 1) [38].

#### 2.5.3. Potential Ecological Risk Index Method (*ERI*)

The *ERI* and *RI* (comprehensive potential ecological risk index) were put forward by Swedish scientist Hakanson (1980). According to the content, type, toxicity level, environmental response, and water sensitivity to heavy metal pollution in sediments, the pollution degree of heavy metals in sediments was evaluated [39]. The following formula is used to calculate:

(3) $${ERI=T}_{r}^{i}\times \frac{C^{i}}{C_{n}^{i}}$$

(4) $$RI=\Sigma_{1}^{i}ERI=\Sigma_{1}^{i}T_{r}^{i}\times \frac{C^{i}}{C_{n}^{i}}$$

where *ERI* is the potential ecological risk index of a single heavy metal; $T_{r}^{i}$ is the toxicity coefficient of the i-th heavy metal in the sediments, and the toxicity coefficients of Cu, Ni, Zn, Pb, Cr, and Cd are 5, 5, 1, 5, 2, and 30, respectively [40,41]. $C^{i}$ represents the measured value of the i-th heavy metal; $C_{n}^{i}$ represents the background value of the i-th heavy metal; *RI* is the comprehensive potential ecological risk index. See Table 1 for the evaluation standard and grading level of *ERI* and *RI* [42].

#### 2.5.4. Sediment Quality Guidelines Values (SQGs)

The National Oceanic and Atmospheric Administration (NOAA) of the United States has formulated many SQGs to represent the relevant critical chemical levels with or without biological impact on the area, which have been widely used to evaluate the biological effects of heavy metals in river sediments [43]. In this paper, threshold effect concentration (TEC)–probable effect concentration (PEC) is selected as the benchmark value of sediment quality.

TEC and PEC are universally recognized sediment quality standards, which are calculated by using the existing SQGs [44]. TEC is the geometric average of threshold effect content category data in other benchmarks; PEC is the geometric average of the possible effect content category data in other benchmarks. When the content of heavy metals in the sediments of a water body is lower than TEC, it indicates that the biotoxicity effect may rarely occur. When the content of heavy metals in river sediment is higher than PEC, it indicates that the biotoxicity effect may often be observed. Among them, the mean PEC quotient (*M*-*PEC*-*Q*) is often used to determine the potential ecological risk of multiple heavy metals to the quality of sediment [45]. The specific calculation formula is as follows:

(5) $$M\text{-}PEC\text{-}Q=\frac{\Sigma_{i=1}^{n}\frac{C_{i}}{PEC_{i}}}{n}$$

where $C_{i}$ is the content of heavy metals in sediments, $PEC_{i}$ is the corresponding *PEC* value of heavy metals, and *n* represents the number of heavy metals. When *M*-*PEC*-*Q* < 0.5, it shows that the sediment sample is non-toxic or has no toxic effect on organisms; when *M*-*PEC*-*Q* > 1.5, it shows that the sediment samples are toxic or have toxic effects on organisms.

### 2.6. Multivariate Statistical Analysis Method

Multivariate statistical methods such as regression analysis, correlation analysis, principal component analysis, and cluster analysis are often used to determine the sources of heavy metals [46] at present. This paper mainly uses correlation coefficient analysis and principal component analysis, two widely used multivariate statistical methods, to determine the sources of heavy metals.

#### 2.6.1. Correlation Coefficient Analysis

The correlation coefficient of heavy metals was employed to identify their sources. Generally speaking, the stronger the correlation between heavy metals, the more likely they are to share the same source. Correlation coefficient analysis is a method used to determine whether heavy metals have the same source based on the degree of correlation between them. If the degree of correlation is high and a significant correlation exists, it is considered that the two heavy metals originate from the same source [47].

#### 2.6.2. Principal Component Analysis (*PCA*)

*PCA* is a classic and highly effective multivariate statistical method for identifying the sources of heavy metals in sediments. This method leverages the correlations among heavy metals to reduce the dimensionality of the original heavy metal data and combine them into several uncorrelated components. These reduced components are then used to reflect the information of the original heavy metals [48]. In this paper, heavy metals that fall within the same component are regarded as having similar information and are thus considered to have similar sources.

## 3. Results and Discussion

### 3.1. Contents of Heavy Metals

The average, maximum, minimum, standard deviation (SD), and coefficient of variation (CV) of eight heavy metals in 226 surface sediment samples in the mid–upper reaches of the Yellow River were calculated, and the sampling points were compared with the average heavy metal content of soil, upper crust [49], and shale [50] (Table 2).

**Figure 2:**
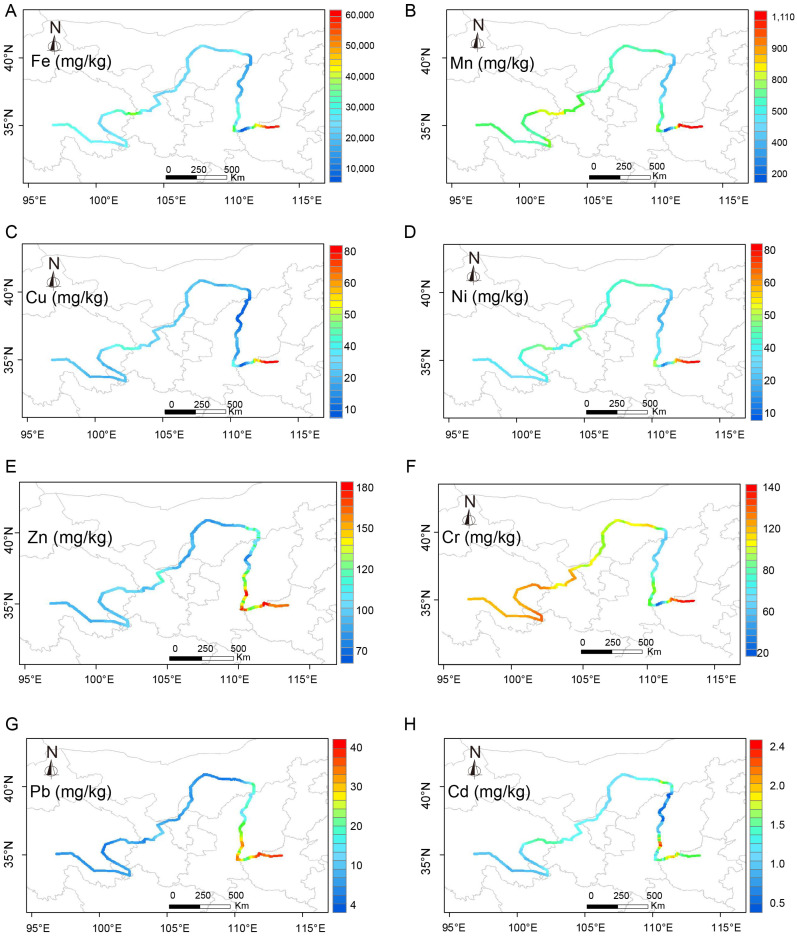
Spatial Distribution of heavy metal in sediments: (**A**) Fe, (**B**) Mn, (**C**) Cu, (**D**) Ni, (**E**) Zn, (**F**) Cr, (**G**) Pb, and (**H**) Cd in mid–upper reaches of the Yellow River. The local map of China in the image is from the website of Chinese National Geography (website: http://www.dili360.com/, accessed on 20 December 2023).

The average mass ratios of heavy metals such as Fe, Mn, Cu, Ni, Cr, Pb, and Cd all exceeded the corresponding average value of onshore soil except for heavy metal Zn. In particular, the average content of Cr was 2.61 times this average. The average contents of heavy metals Mn, Ni, Zn, Cr, and Cd were higher than average value of sediments in China (Mean—sediments), where Cr was 2.67 times higher, while Cd was 12.3 times higher. Only the average contents of Zn, Cr, and Cd exceeded the background value, which were 1.16 times, 1.04 times, and 4.1 times the value, respectively. The average content of Cd is relatively high compared with the average content of heavy metals in the crust (background value) adopted in this paper (Table 2). This showed that Cr and Cd were greatly influenced by human activities and accumulated obviously, and they were the main pollution factors in the study area. The coefficient of variation of eight heavy metals ranged from 43.9% to 91.9%, all of which reached a high degree of variation (CV value of medium degree of variation ranged from 15% to 36%), indicating that there were significant differences in the spatial distribution of each heavy metal mass ratio, and this conclusion could also be confirmed from the spatial distribution map (Figure 2).

### 3.2. Spatial Distribution Characteristics

ArcGIS 10.7 software was used for the distribution of the content of heavy metals in the sediment of the mid–upper reaches of the Yellow River. The content was interpolated by kriging, the buffer zone of the mid–upper reaches of the Yellow River was constructed, and the spatial distribution map of each heavy metal was finally obtained through mask extraction (Figure 2). Due to the complex regional environment and the diversity of human activities on both sides of the Yellow River, there were some similarities and differences in the spatial distribution of heavy metals in the sediment of the mid–upper reaches of the Yellow River. All the heavy metals were in the joint area between Gansu Province and Qinghai Province (sample points 20 to 23), Weinan City in Shaanxi Province (sites 123 to 144), Baotou City in Inner Mongolia (sites 88 to 100), and Luoyang City in Henan Province (sites 212 to 226). The reason for the formation of a high-value area between Gansu Province and Qinghai Province might be due to the poor water quality of Huangshui River flowing through here, as well as the discharge of various sewage in Xining City and Haidong City. As Weinan was a heavy industrial city, the Weihe River, the largest tributary of the Yellow River that ran through the city, was seriously polluted by industrial emissions. In addition, there are many other tributaries in the suburbs of Weinan, and agricultural production and domestic sewage was discharged into the Yellow River [51]. The main reason for the formation of the high-value area in Baotou City, Inner Mongolia might be the use of chemical fertilizers and pesticides in the agricultural production process [52]; the formation of the high-value area in Luoyang City, Henan Province was also due to the combination of the above reasons. Relatively low values of heavy metals Fe, Cu, Ni, Zn, Pb, and Cd were found in the watershed of Qinghai Province (samples 1 to 7), the southern part of Gannan Prefecture of Gansu Province (samples 7 to 10), and Yan’an City and Yulin City of Shaanxi Province (samples 151 to 186), which might be due to the mountainous landscape, a smaller population, and fewer human activities. Heavy metals were mainly caused by rock weathering.

Heavy metals Cu, Fe, Mn, Ni, Zn, Pb, and Cr all reached the highest value in Luoyang City. The overall distribution of Fe, Cu, Ni, Zn, and Pb has not changed much, and all of them cause high pollution in the southeast of Weinan City and Luoyang City, while other river sections have less pollution; Cr and Mn were found except in Yan’an City and Yulin City, and both metals reach the highest value in Luoyang City, while Cr was mainly distributed in higher-value categories as a whole. Heavy metal Cd was distributed sporadically in all sections of the basin, and there were relatively high values at individual points in the area where Gansu Province meets Qinghai Province, Baotou City, Weinan City, Sanmenxia City, and Luoyang City. On the whole, the distribution of heavy metal Cr was mainly in the higher-value category of 110–150 mg/kg.

### 3.3. Assessment of Heavy Metal Pollution

#### 3.3.1. EFs

**Figure 3:**
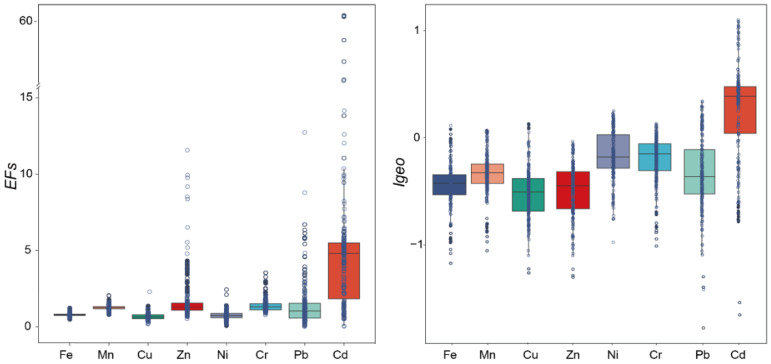
Boxplots of EFs and I_geo_s of heavy metals in surface sediments from the mid–upper reaches of the Yellow River.

The ranking of the average EFs of heavy metals in the sediment of the mid–upper reaches of the Yellow River was Cd > Zn > Pb > Cr > Mn > Fe > Ni > Cu, and the value of the comprehensive average was 1.81, ranging from 1 to 3, which belongs to the micro-enrichment level as a whole (Table 1 and Table 3). The average EFs of Fe, Ni, and Cu are all less than 1, indicating that they are not enriched in the sediment of the mid–upper reaches of the Yellow River, and the average EFs of Mn, Zn, Cr, and Pb are all between 1 and 3, indicating that they are slightly enriched in the sediment of the mid–upper reaches of the Yellow River. Cd has the highest enrichment degree, with an average EF value of 6.32, which belongs to heavy enrichment, and 107 points in the mid–upper reaches of the Yellow River have reached a heavy enrichment level, followed by a few points (9 and 10) with Zn and Pb, indicating that these heavy metals are more affected by human beings. The enrichment of Fe, Mn, Cu, Ni, and Cr is low, and they are all at the level of no enrichment or micro-enrichment, indicating that these heavy metals are less influenced by human activities and mainly come from nature [53] (Figure 3).

Fe, Mn, Cu, and Cr were moderately variable (CV = 15–36%), while Zn, Ni, Pb, and Cd were highly variable (CV > 36%), indicating that the enrichment areas of these heavy metals were scattered in the mid–upper reaches of the Yellow River (Table 3).

#### 3.3.2. I_geo_

The average I_geo_s of heavy metals in the sediments of the mid–upper reaches of the Yellow River were in the order of Cd (0.73) > Cr (0.21) > Ni (0.05) > Zn (−0.04) > Mn (−0.18) > Cu (−0.27) > Fe (−0.3) > Pb (−0.34) (Table 1 and Table 4). The average I_geo_ values of Fe, Mn, Cu, Zn, and Pb were all less than 1, indicating no pollution; the average I_geo_ values of Cd, Cr, and Ni were between 0 and 1, indicating slight pollution. The value of the comprehensive average was −0.29, indicating that the mid–upper reaches of the Yellow River were not polluted by heavy metals.

Among the 226 sample sites studied, the I_geo_s of eight kinds of heavy metals of 132 sample sites were less than 0, accounting for the majority, and there were 89 sample sites with I_geo_s between 0 and 1, indicating light pollution of heavy metals at the corresponding sites, of which Ni (143), Cr (195), and Cd (168) have more sites corresponding to light pollution, while Cd has 37 sites between 1 and 2, which indicates moderate pollution (Figure 3).

The variation coefficients of eight kinds of heavy metals were all strong, of which the variation coefficients of Mn, Ni, Zn, Cr, and Pb were 111.11%, 500.00%, 575.00%, 104.76%, and 105.88%, with values over 100% indicating that the sites polluted by these heavy metals were relatively scattered and seriously affected by human production activities on both sides (Table 4).

#### 3.3.3. Potential Ecological Risk Assessment

The ERI of Cu, Ni, Zn, Cr, Pb, and Cd in the surface sediments of the mid–upper reaches of the Yellow River ranged from 0. 09 to 10.2, 0.38 to 6. 93, 0.01 to 2. 68, 0.03 to 10.2, 0.02 to 6. 59, and 1 to 567, respectively. The order of ERI values of heavy metals from big to small was Cd (123) > Cr (5.2) > Ni (2.84) > Cu (2.62) > Pb (1.86) > Zn (1.16). All of them are less than 40 (low risk) except for that of Cd, whereas the RI value reached 137 after adding Cd. The maximum ERI value of Cd was 567, which was greater than 320, indicating that Cd has a strong potential ecological risk in some sampling sites of the mid–upper reaches of the Yellow River. However, the overall ecological pollution level of eight kinds of heavy metals was of medium risk (Table 1 and Table 5).

#### 3.3.4. SQGs

The contents of heavy metals Cu, Zn, and Pb in the sediments were lower than those of 82.7%, 68.1%, and 82.3% of the total sample points of the mid–upper reaches of the Yellow River, while those between TEC and PEC account for 17.3%, 31.9%, and 17.7% of the total sample points, and there was no sample point higher than PEC, indicating that these three heavy metals have basically no biotoxicity effect in the mid–upper reaches of the Yellow River. The samples with Ni, Cr, and Cd contents lower than their TEC accounted for 27%, 8.85%, and 38.1% of the total samples, while the samples with values between their TEC and PEC accounted for 46.5%, 59.3% and 59.7% of the total samples, and the samples with higher PEC accounted for 26.6% and 31.9% of the total samples, respectively. It shows that, among the 226 samples studied in the mid–upper reaches of the Yellow River, there were very few samples with no biotoxicity risk, and more than half of the samples with risk, 60, 72, and 5 samples, respectively, were likely to have biotoxicity risk from time to time.

The PEC-Q of each heavy metal was calculated to further determine the toxicity risk of heavy metals in the surface sediments of the mid–upper reaches of the Yellow River (Table 6). The mean PEC-Q values of six heavy metals were in the order of Cr (0.84) > Ni (0.79) > Cd (0.25) > Zn (0.24) > Cu (0.16) > Pb (0.14), with an average value of 0.4 and all less than 1.5, indicating that the heavy metals studied were Cu, Ni, and Pb. Of the maximum PEC-Q of each heavy metal, the maximum values of Ni and Cr were 1.94 and 1.65, both exceeding 1.5, indicating that there was a risk of biotoxicity of these two heavy metals in some samples in the mid–upper reaches of the Yellow River.

### 3.4. Source Identification

#### 3.4.1. Correlation Coefficient

According to the Pearson correlation coefficient (Table 7), the correlation coefficients between Fe, Mn, Cu, Ni, and Cr were relatively large at the 0.01 confidence level, indicating that these heavy metals have the same source; meanwhile, Cr was not correlated with Pb and Cd, indicating that Pb and Cd have different sources; Fe, Mn, Cu, Ni and Cr were widely distributed in the natural environment, and they were all considered to come from natural processes, while Pb and Cd were all produced in human production processes [54,55]. The correlation coefficients between Zn and Fe, Mn, Cu, Ni, and Cr were small, indicating that Zn has a low degree of correlation, and a small amount of Zn might come from natural sources. The correlation coefficients between Zn and Pb, as well as between Zn and Cd, were relatively large, and there was a medium-to-high degree of correlation in both cases. It was possible that most of the Zn in the middle and upper reaches of the Yellow River originated from anthropogenic sources.

Cd has a low degree of correlation with Fe, Mn, and Cu at the confidence level of 0.05 but has no correlation with Cr and Pb, indicating that Cd and Pb come from different artificial sources.

#### 3.4.2. Principal Component Analysis

**Figure 4:**
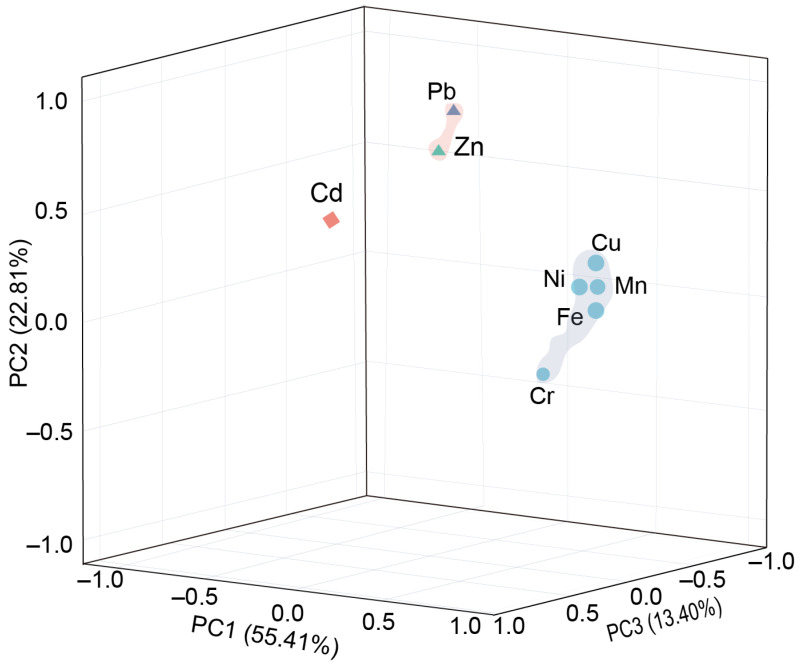
Principal component loading plot for metal variables.

The factor load matrix was obtained by orthogonal rotation with maximum variance, and three principal component factors with eigenvalues greater than 1 were extracted, with a cumulative variance contribution rate of 84.90%, indicating that these three principal component factors can represent the information of the original data (Table 8 and Figure 4).

The variance contribution rate of principal component 1 (PC1) was 55.41%. Among the eight heavy metals, the loads of Fe, Mn, Cu, Ni, and Cr were all relatively high, which were 0.91, 0.94, 0.87, 0.81, and 0.87, respectively, indicating that the above five heavy metals have the same source and mainly come from nature. Therefore, PC1 represents the natural source and was mainly influenced by the local soil-forming parent. The variance contribution rate of principal component 2 (PC2) was 22.81%, and the factor loads of Zn (0.81) and Pb (0.91) were high, which indicated that Zn and Pb come from the same sources, mainly man-made sources. Therefore, PC2 mainly represents man-made sources. The factor loading of Cd was 0.55, which was moderate, indicating that Cd comes from man-made pollution to a great extent. The variance contribution rate of principal component 3 (PC3) was 13.40%, with Cd having a strong load (0.79), and the loads of other metal elements were low, which also showed that Cd mainly comes from man-made sources. For principal component 2 (PC2), Cd comes from different man-made sources compared with Zn and Pb.

The results obtained from the correlation coefficient analysis and principal component analysis were quite similar when it came to identifying the sources of the eight heavy metals in the mid–upper reaches of the Yellow River.

## 4. Conclusions

In conclusion, this study focused on the heavy metal pollution in the surface sediments of the mid–upper reaches of the Yellow River. Through field sampling and laboratory analysis, the concentrations and spatial distribution characteristics of eight heavy metals (Fe, Mn, Cu, Ni, Zn, Cr, Pb, and Cd) were determined. The results show significant differences in the spatial distribution of heavy metals between the upper and middle reaches of the Yellow River, with relatively high levels of Cr and Cd, whose spatial distribution is significantly influenced by human activities. Pollution assessments conducted using various methods indicate a slight accumulation of heavy metals, with Cd presenting a relatively high potential ecological risk. Source identification results show that Fe, Mn, Cu, Ni, and Cr mainly originate from natural sources, while Pb and most of the Zn come from human activities. This study provides important information for understanding the heavy metal pollution status of the Yellow River and has guiding significance for local pollution control and prevention strategies.

## Figures and Tables

**Table 1:** Classification standard of *EF*, *I_geo_*, and *ERI*.

*EF* Classes	Enrichment Level	*I_geo_* Value	Pollution Level	*ERI* Classes	*RI* Classes	Ecological Risk Level
*EF* < 1	No enrichment	*I_geo_* ≤ 0	No pollution	——	——	——
1 ≤ *EF* < 3	Micro-enrichment	0 < *I_geo_* ≤ 1	Light pollution	*ERI* < 40	*RI* < 95	Low risk
3 ≤ *EF* < 5	Medium enrichment	1 < *I_geo_* ≤ 2	Moderate pollution	40 ≤ *ERI* < 80	95 ≤ *RI* < 190	Medium risk
5 ≤ *EF* < 10	Heavier enrichment	2 < *I_geo_* ≤ 3	Heavy pollution	80 ≤ *ERI* < 160	190 ≤ *RI* < 380	High risk
10 ≤ *EF* < 25	Strong enrichment	3 < *I_geo_* ≤ 4	Severe pollution	160 ≤ *ERI* < 320	*RI* ≥ 380	Extra-high risk
25 ≤ *EF* < 50	Extremely rich	4 < *I_geo_* ≤ 5	Serious pollution	*ERI* ≥ 320	——	Extremely risky

**Table 2:** Statistics of heavy metal content in sediments of the mid–upper reaches of the Yellow River.

Heavy Metal (mg/kg)	Fe	Mn	Cu	Ni	Zn	Cr	Pb	Cd
Mean	28,800	649	23.7	38.6	111	93.8	18.4	1.23
Minimum	4760	113	3.70	5.10	15.1	13.2	0.500	0.0100
Maximum	96,700	1490	91.4	94.3	255	183	65.9	5.67
Standard deviation (SD)	13,900	285	14.6	19.8	55.5	37.9	14.5	1.13
Coefficient of variation (CV%)	48.3	43.9	61.5	51.4	50.1	40.4	78.6	91.9
Mean—soil	24,000	494	17.0	28.5	193	35.9	15.9	0.890
Mass ratio of sediment to soil	1.20	1.31	1.39	1.35	0.570	2.61	1.16	1.38
Mean—upper crust	35,000	600	25.0	20.0	71.0	35.0	20.0	0.100
Mean—shale	47,200	850	45.0	68.0	95.0	90.0	20.0	0.300

**Table 3:** Descriptive statistics of EF of heavy metals in sediments from the mid–upper reaches of the Yellow River (sample number = 226, unit: mg/kg).

Statistical Values	Fe	Mn	Cu	Ni	Zn	Cr	Pb	Cd	Mean
Minimum	0.49	0.80	0.01	0.08	0.01	0.01	0.01	0.04	0.18
Maximum	1.25	2.03	2.36	2.45	11.7	3.55	12.8	69.4	13.2
Mean	0.80	1.27	0.68	0.76	1.88	1.39	1.43	6.32	1.81
Standard deviation (SD)	0.11	0.16	0.23	0.29	1.78	0.41	1.54	8.21	1.59
Coefficient of variation (CV%)	13.8	12.6	33.82	38.2	94.7	29.5	107.7	130.0	57.5
Background value	47,200	850	45.0	68.0	95.0	90.0	20.0	0.30	6046
*EF* < 1 (individual)	214	12	217	190	34	12	112	27	102
1 ≤ *EF* < 3 (individual)	12	214	9	36	166	211	95	44	98
3 ≤ *EF* < 5 (individual)	0	0	0	0	17	3	10	48	10
*EF* ≥ 5 (individual)	0	0	0	0	9	0	10	107	16

**Table 4:** Descriptive statistics of I_geo_s of heavy metals in the sediments from the mid–upper reaches of the Yellow River (sample number = 226, unit: mg/kg).

Statistical Values	Fe	Mn	Cu	Ni	Zn	Cr	Pb	Cd	Mean
Minimum	−1.04	−0.900	−1.01	−0.770	−0.850	−0.600	−1.78	−1.18	−2.21
Maximum	0.270	0.220	0.390	0.500	0.380	0.540	0.340	1.58	0.270
Mean	−0.300	−0.180	−0.270	0.0500	−0.0400	0.210	−0.340	0.730	−0.290
Standard deviation (SD)	0.200	0.200	0.240	0.250	0.230	0.220	0.360	0.470	0.300
Coefficient of variation (CV%)	66.7	111	88.9	500	575	105	106	64.4	202
Background value	35,000	600	25.0	20.0	71.0	35.0	20.0	0.100	4470
*I_geo_* ≤ 0 (individual)	209	196	202	83	138	31	180	21	132
0 < *I_geo_* ≤ 1 (individual)	17	30	24	143	88	195	46	168	89
1 < *I_geo_* ≤ 2 (individual)	0	0	0	0	0	0	0	37	5

**Table 5:** Statistics of ERI and RI of heavy metals in surface sediments from the mid–upper reaches of the Yellow River.

			ERI				RI
	Cu	Ni	Zn	Cr	Pb	Cd	
Minimum	0.0900	0.380	0.0100	0.0300	0.0200	1.00	1.53
Maximum	10.2	6.93	2.68	10.2	6.59	567	604
Mean	2.62	2.84	1.16	5.20	1.84	123	137
Grade	Low risk	Low risk	Low risk	Low risk	Low risk	Medium risk	Medium risk

**Table 6:** Statistics of SQGs (TEC-PEC) for heavy metals from the mid–upper reaches of the Yellow River.

SQGs	Cu	Ni	Zn	Cr	Pb	Cd
TEC	31.6	22.7	121	43.4	35.8	0.990
PEC	149	48.6	459	111	128	4.98
<TEC (%)	82.7	27.0	68.1	8.85	82.3	38.1
TEC (%)–PEC (%)	17.3	46.5	31.9	59.3	17.7	59.7
>PEC (%)	0.00	26.6	0.00	31.9	0.00	2.21
Mean PEC-Q	0.16	0.79	0.24	0.85	0.14	0.25
Minimal PEC-Q	0.0250	0.105	0.0330	0.119	0.00400	0.00200
Maximal PEC-Q	0.610	1.94	0.550	1.65	0.520	1.14

**Table 7:** Correlation coefficients between different heavy metal elements (*n* = 226).

Metal	Fe	Mn	Cu	Ni	Zn	Cr	Pb	Cd
Fe	1							
Mn	0.921 **	1						
Cu	0.823 **	0.820 **	1					
Ni	0.668 **	0.707 **	0.717 **	1				
Zn	0.386 **	0.399 **	0.400 **	0.284 **	1			
Cr	0.729 **	0.796 **	0.657 **	0.601 **	0.222 **	1		
Pb	0.346 **	0.335 **	0.351 **	0.239 **	0.680 **	0.080	1	
Cd	0.143 *	0.133 *	0.142 *	0.079	0.564 **	0.107	0.569 **	1

Note: Levels of significance: * *p* < 0.05; ** *p* < 0.01.

**Table 8:** Total variance explained and rotated component matrix of principal component analysis.

Metal	PC1	PC2	PC3
Fe	0.91	0.22	0.04
Mn	0.94	0.19	0.07
Cu	0.87	0.28	−0.06
Ni	0.81	0.18	−0.13
Zn	0.25	0.81	0.25
Cr	0.87	−0.15	0.34
Pb	0.15	0.91	0.09
Cd	−0.01	0.55	0.79
Eigenvalue	3.95	2.02	0.83
Variance contribution rate (%)	55.41	22.81	6.68
Cumulative variance contribution rate (%)	55.41	78.22	84.90

## Data Availability

The datasets used and/or analyzed during the current study are available from the corresponding author on reasonable request.

## Supplementary Materials

The following supporting information can be downloaded at: https://www.mdpi.com/article/10.3390/toxics13030150/s1.
