# Supplementary material for: Assessment of Heavy Metal Contamination, Distribution, and Source Identification in Surface Sediments from the Mid–Upper Reaches of the Yellow River
Source: Toxics. 2025 Feb 23;13(3):150. doi: 10.3390/toxics13030150 (PMC11945980; doi:10.3390/toxics13030150)
Supplement: Supplementary file 1 [file toxics-13-00150-s001.zip › toxics-3464628-supplementary.pdf]

Sampling locations in Henan, Shaanxi, Gansu, Inner Mongolia, Ningxia and Qinghai provinces（unit：mg/kg）			
**Number**	**Longitude/°**	**Latitude/°**	**Specific sampling location**
HN1	110.43	34.59	Lingbao City, Sanmenxia City, Henan Province
HN2	110.55	34.58	Lingbao City, Sanmenxia City, Henan Province
HN3	110.68	34.60	Lingbao City, Sanmenxia City, Henan Province
HN4	110.76	34.65	Lingbao City, Sanmenxia City, Henan Province
HN5	110.83	34.62	Near Dongguyi Scenic Area, Lingbao City, Sanmenxia City, Henan Province
HN6	110.92	34.71	Yunbao Yellow River Bridge, Lingbao City, Sanmenxia City, Henan Province
HN7	111.01	34.73	Near Fengzuodukou, Lingbao City, Sanmenxia City, Henan Provinc
HN8	111.06	34.77	He Jia Tan Cun, Pinglu County, Yuncheng City, Shanxi Province
HN9	111.09	34.74	Near Chengcun Forestry Team, Shanzhou District, Sanmenxia City, Henan Province
HN10	111.10	34.74	Near Tiantong Mining Co., Ltd., Shanzhou District, Sanmenxia City, Henan Province
HN11	111.11	34.79	Bei Village, Pinglu County, Yuncheng City, Shanxi Province
HN12	111.16	34.81	Yellow River Story Theme Park, Hubin District, Sanmenxia City, Henan Province
HN13	111.20	34.80	Huanghe Park, Hubin District, Sanmenxia City, Henan Provinc
HN14	111.23	34.80	Pinglu County, Yuncheng City, Shanxi Province

HN15	111.17	34.81	Hubin District, Sanmenxia City, Henan Province
HN16	111.21	34.79	Near Ludang Yanyu Lake Scenic Area, Hubin District, Sanmenxia City, Henan Province
HN17	111.24	34.77	Hubin District, Sanmenxia City, Henan Province
HN18	111.26	34.80	Sandalu Road, Hubin District, Sanmenxia City, Henan Provinc
HN19	111.32	34.82	Sandalu Road, Hubin District, Sanmenxia City, Henan Provinc
HN20	111.59	34.82	Mianchi County, Sanmenxia City, Henan Province
HN21	111.60	34.89	Near Bailang Village Committee, Mianchi County, Sanmenxia City, Henan Province
HN22	111.73	34.89	Mianchi County, Sanmenxia City, Henan Province
HN23	111.80	35.03	yuanqu County, Yuncheng City, Shanxi Province
HN24	111.81	35.07	yuanqu County, Yuncheng City, Shanxi Province
HN25	111.82	35.06	yuanqu County, Yuncheng City, Shanxi Province
HN26	112.19	34.93	Near Taoyuan, Xin'an County, Luoyang City, Henan Provinc
HN27	112.24	34.93	Near Hexi Village, Xin'an County, Luoyang City, Henan Provinc
HN28	112.20	34.97	Shizu Mountain Scenic Spot, Wanshan Lake, Xin'an County, Luoyang City, Henan Provinc
HN29	112.21	34.97	Niushi line, Xin'an county, Luoyang City, Henan province
HN30	112.36	34.90	Mengjin District, Luoyang City, Henan Province

HN31	112.35	34.91	Mengjin District, Luoyang City, Henan Province
HN32	112.66	34.85	Mengjin County, Mengjin District, Luoyang City, Henan Provinc
HN33	112.62	35.02	Jiyuan City, Henan Province
HN34	112.59	34.85	Zhoukou Village, Mengjin District, Luoyang City, Henan Provinc
HN35	112.58	34.89	Near the Forest Farm of Mengjin District, Luoyang City, Henan Province
HN36	112.54	34.88	Xixiayuan scenic spot, Mengjin district, Luoyang City, Henan province
HN37	112.50	34.90	Near Wangzhuang Village, Mengjin District, Luoyang City, Henan Provinc
HN38	112.48	34.90	Near Shilin Village, Mengjin District, Luoyang City, Henan Provinc
HN39	113.45	34.92	Near the channel along the Yellow River in Xingyang City, Zhengzhou City, Henan Province
HN40	112.45	34.92	Near S95 (Jiluo Expressway), Mengjin District, Luoyang City, Henan Province
SX1	110.29	34.61	Near G30 (Lianhuo Expressway), Tongguan County, Weinan City, Shaanxi Province
SX2	110.19	34.62	Huayin City, Weinan City, Shaanxi Province
SX3	110.23	34.73	Dali County, Weinan City, Shaanxi Province
SX4	110.23	34.80	Dali County, Weinan City, Shaanxi Province
SX5	110.21	34.92	Dali County, Weinan City, Shaanxi Province
SX6	110.21	34.97	Dali County, Weinan City, Shaanxi Province

SX7	110.24	34.99	Heyang County, Weinan City, Shaanxi Province
SX8	110.28	35.00	Heyang County, Weinan City, Shaanxi Province
SX9	110.32	35.09	Heyang County, Weinan City, Shaanxi Province
SX10	110.37	35.14	Near Qiachuan National Scenic Spot, Heyang County, Weinan City, Shaanxi Province
SX11	110.35	35.18	China Qiachuan Shenquan Scenic Spot, Heyang County, Weinan City, Shaanxi Province
SX12	110.36	35.21	Taili Village, Heyang County, Weinan City, Shaanxi Province
SX13	110.38	35.28	Xiayuantou, Heyang County, Weinan City, Shaanxi Province
SX14	110.40	35.32	Near Yanhuang Sightseeing Road, Heyang County, Weinan City, Shaanxi Province
SX15	110.41	35.37	Near G5 (Xiyu Expressway), Hancheng City, Weinan City, Shaanxi Province
SX16	110.47	35.45	Hancheng, Weinan City, Shaanxi Province
SX17	110.49	35.49	Zhouyuan Village Committee, Hancheng City, Weinan City, Shaanxi Province
SX18	110.51	35.53	Qian Ma Ke Cun, Hancheng City, Weinan City, Shaanxi Provinc
SX19	110.54	35.55	Kui Village, Hancheng City, Weinan City, Shaanxi Province
SX20	110.59	35.61	Zhongxin Village, Hancheng City, Weinan City, Shaanxi Province
SX21	110.60	35.66	Near Longmen Tunnel, Hancheng City, Weinan City, Shaanxi Province
SX22	110.57	35.75	Near Leidong Bridge, Hancheng City, Weinan City, Shaanxi Province

SX23	110.53	35.88	Yanhuang Sightseeing Road, Mashuping, Yichuan County, Yan'an City, Shaanxi Province
SX24	110.50	35.98	Laojibao, Yichuan County, Yan'an City, Shaanxi Province
SX25	110.47	36.05	Yanhuang Sightseeing Road, Yichuan County, Yan'an City, Shaanxi Province
SX26	110.48	36.13	Near Hukou Scenic Spot of the Yellow River in Ji County, Linfen City, Shanxi Province
SX27	110.48	36.18	Ji County, Linfen City, Shanxi Province
SX28	110.47	36.28	Near Xiawan, Yichuan County, Yan'an City, Shaanxi Province
SX29	110.47	36.41	Yanchang County, Yan'an City, Shaanxi Province
SX30	110.49	36.50	Yanchang County, Yan'an City, Shaanxi Province
SX31	110.48	36.61	Lijiashan, Yonghe County, Linfen City, Shanxi Province
SX32	110.41	36.70	Near Jinshan Gorge, Yonghe County, Linfen City, Shanxi Province
SX33	110.41	36.81	Gaojiapan Village, Yanchuan County, Yan'an City, Shaanxi Province
SX34	110.39	36.91	Liu Jia Pan Cun, Yanchuan County, Yan'an City, Shaanxi Province
SX35	110.44	37.05	Near Wangjiahe Village, Qingjian County, Yulin City, Shaanxi Province
SX36	110.59	37.19	Near Hanjiashan Village, Qingjian County, Yulin City, Shaanxi Province
SX37	110.68	37.29	Near the Village Committee of Changli Village, Liulin County, Luliang City, Shanxi Province
SX38	110.67	37.39	Hu Jia Ta Cun, Liulin County, Lvliang City, Shanxi Province

SX39	110.75	37.48	Yanhuang Highway, Wubao County, Yulin City, Shaanxi Province
SX40	110.79	37.65	Yellow River Dongdu Scenic Area, Wubao County, Yulin City, Shaanxi Province
SX41	110.75	37.76	Rangou Village, Jia County, Yulin City, Shaanxi Province
SX42	110.61	37.89	Ye Ze Ping, Jia County, Yulin City, Shaanxi Province
SX43	110.50	38.01	Near Jiaxian White Cloud Mountain Scenic Spot, Yulin City, Shaanxi Province
SX44	110.51	38.12	Da Ping Village, Jia County, Yulin City, Shaanxi Province
SX45	110.57	38.25	Fuxing Bay, Shenmu City, Yulin City, Shaanxi Province
SX46	110.65	38.31	Xi Dou Yu Cun, Shenmu City, Yulin City, Shaanxi Provinc
SX47	110.76	38.41	Xia Wang Jia Ping Cun, Shenmu City, Yulin City, Shaanxi Provinc
SX48	110.87	38.49	Yanhuang Road, Shenmu City, Yulin City, Shaanxi Province
SX49	110.88	38.61	Near Mapan Road, Shenmu City, Yulin City, Shaanxi Province
SX50	110.95	38.74	Near Oolong Mountain Scenic Area, Fugu County, Yulin City, Shaanxi Province
SX51	111.01	38.88	Xishantou Village, Baode County, Xinzhou City, Shanxi Province
SX52	111.11	39.05	Fuqiang Road, Fugu County, Yulin City, Shaanxi Province
SX53	111.16	39.14	Langwan, Fugu County, Yulin City, Shaanxi Province
SX54	111.22	39.25	Huangfu Town, Fugu County, Yulin City, Shaanxi Province

SX55	111.12	39.36	Xiaozhan, Zhungeer Banner, Ordos City, Inner Mongolia Autonomous Region
SX56	111.26	39.44	Haomigetuo, Zhungeer Banner, Ordos City, Inner Mongolia Autonomous Region
SX57	111.41	39.49	No.1 Yellow River Tourist Highway, Pianguan County, Xinzhou City, Shanxi Province
SX58	111.43	39.57	Jianshanzi, S103, Zhungeer Banner, Ordos City, Inner Mongolia Autonomous Region
SX59	111.41	39.69	Shata, Zhungeer Banner, Ordos City, Inner Mongolia Autonomous Region
SX60	111.41	39.84	Aobaoshang, Zhungeer Banner, Ordos City, Inner Mongolia Autonomous Region
SX61	111.44	39.96	Yanhuang Road, Qingshuihe County, Hohhot City, Inner Mongolia Autonomous Region
SX62	111.41	40.07	Qingshuihe County, Hohhot City, Inner Mongolia Autonomous Region
SX63	111.34	40.15	Y102, Tuoketuo County, Hohhot City, Inner Mongolia Autonomous Region
SX64	111.20	40.23	Tuoketuo County, Hohhot City, Inner Mongolia Autonomous Region
GS1	104.38	37.18	Nangoutai, Yihe Village, Shuanglong Town, Jingyuan County, Gansu Provinc
GS2	103.55	36.11	Dongchuan Town, Xigu District, Lanzhou City, Gansu Provinc
GS3	103.56	36.12	Liu Quan Zhen An Men Cun, Xigu District, Lanzhou City, Gansu Provinc
GS4	103.61	36.14	Sha Jing Yi street, Anning District, Lanzhou City, Gansu Provinc
GS5	103.69	36.09	Near Nongda, Anning District, Lanzhou City, Gansu Provinc
GS6	103.73	36.09	Party School of Anning District, Lanzhou City, Gansu Provinc

GS7	103.77	36.08	Under Qilihe Bridge, Lanzhou City, Gansu Provinc
GS8	103.80	36.07	Xiaoxihu, Lanzhou City, Gansu Province
GS9	103.86	36.07	Yantan Bridge, Lanzhou City, Gansu Provinc
GS10	103.93	36.05	Donggang Yellow River Bridge, Lanzhou City, Gansu Provinc
GS11	104.02	36.06	Lai Zi Bao Xiang Dong Ping Cun, Yuzhong County, Gansu Provinc
GS12	104.00	36.17	Yellow River Suspension Bridge in Shichuan Town, Gaolan County, Gansu Province
GS13	104.00	36.15	Downstream of Lihua Bridge, Shichuan Town, Gaolan County, Gansu Provinc
GS14	104.24	36.36	Shuichuan Town, Baiyin District, Baiyin City, Gansu Provinc
GS15	104.41	36.43	Si Long Zhen Min Le Cun, Baiyin District, Baiyin City, Gansu Provinc
GS16	104.63	36.57	400m upstream of Yellow River Railway Bridge, Jingyuan County, Gansu Province
GS17	104.68	36.58	Downstream of Yellow River Bridge at Miltan, Jingyuan County, Gansu Province
GS18	104.71	36.60	Wangjiabai, Milan Town, Jingyuan County, Gansu Provinc
GS19	104.31	37.15	Wufo Township, Jingtai County, Gansu Province
GS20	104.29	37.16	Wu Fo Xiang, Jingtai County, Gansu Province
GS21	103.37	36.12	Dachuan Town, Xigu District, Lanzhou City, Gansu Provinc
GS22	103.35	36.11	Jiaojia Village, Yanguoxia Town, Yongjing County, Gansu Provinc

GS23	103.33	36.08	Yanguoxia Town Shangche Village, Yongjing County, Gansu Provinc
GS24	103.22	35.98	Tai Ji Zhen Da Chuan Cun, Yongjing County, Gansu Provinc
GS25	103.31	35.94	Yongjing County, Gansu Province
GS26	103.32	35.90	Dongling Township, Dongxiang Autonomous County, Gansu Province
GS27	102.79	35.86	Chen Jia Cun, Dahejia Town, Jishan County, Gansu Provinc
GS28	102.75	35.84	Dahejia Town, Jishan County, Gansu Province
NM1	111.16	40.23	Shuanghe Town, Tuoketuo County, Inner Mongolia
NM2	111.08	40.26	Shuanghe Town, Tuoketuo County, Inner Mongolia
NM3	110.88	40.26	Jiangjunyao Town, Tumote Right Banner, Baotou City, Inner Mongolia
NM4	110.79	40.27	Jiangjunyao Town, Tumote Right Banner, Baotou City, Inner Mongolia
NM5	110.52	40.38	Mingshanao Township, Tumote Right Banner, Baotou City, Inner Mongolia
NM6	110.51	40.39	Mingshanao Township, Tumote Right Banner, Baotou City, Inner Mongolia
NM7	110.36	40.46	Mingshanao Township, Tumote Right Banner, Baotou City, Inner Mongolia
NM8	110.02	40.53	Donghe District near Baotou City, Inner Mongolia
NM9	109.99	40.51	Jiuyuan District, Baotou City, Inner Mongolia
NM10	109.92	40.53	Jiuyuan District, Baotou City, Inner Mongolia

NM11	109.68	40.52	Machi Town, Jiuyuan District, Baotou City, Inner Mongolia
NM12	109.77	40.51	Haringer Town, Jiuyuan District, Baotou City, Inner Mongolia
NM13	109.75	40.50	Haringer Town, Jiuyuan District, Baotou City, Inner Mongolia
NM14	109.64	40.54	Haringer Town, Jiuyuan District, Baotou City, Inner Mongolia
NM15	109.45	40.52	Ha Ye Hu Tong Zhen, Jiuyuan District, Baotou City, Inner Mongolia
NM16	109.16	40.53	Xianfeng Town, Wulata Front Banner, Bayannur City, Inner Mongolia
NM17	108.88	40.56	Xianfeng Town, Wulata Front Banner, Bayannur City, Inner Mongolia
NM18	108.77	40.61	Xianfeng Town, Wulata Front Banner, Bayannur City, Inner Mongolia
NM19	108.70	40.65	Xianfeng Town, Wulata Front Banner, Bayannur City, Inner Mongolia
NM20	108.02	40.86	Bayantaohai Town, Wuyuan District, Bayanzer City, Inner Mongolia
NM21	107.94	40.87	Bayantaohai Town, Wuyuan District, Bayanzer City, Inner Mongolia
NM22	107.73	40.86	Tianjitai Town, Wuyuan District, Bayanzer City, Inner Mongolia
NM23	107.66	40.79	Tianjitai Town, Wuyuan District, Bayanzer City, Inner Mongolia
NM24	107.44	40.68	Shuanghe Town, Linhe District, Bayannur City, Inner Mongolia
NM25	107.14	40.43	Dukou Town, Dengkou County, Bayannur City, Inner Mongolia
NM26	107.12	40.38	Dukou Town, Dengkou County, Bayannur City, Inner Mongolia

NM27	107.03	40.31	Bayangaole Town, Dengkou County, Bayannur City, Inner Mongolia
NM28	107.02	40.26	Balagong Town, Hangjin Banner, Ordos City, Inner Mongolia
NM29	106.73	39.93	Mengxi Town, Etuoke Banner, Ordos, Inner Mongolia
NM30	106.73	39.92	Mengxi Town, Etuoke Banner, Ordos, Inner Mongolia
NM31	106.76	39.81	Qianlishan Town, Haibowan District, Wuhai City, Inner Mongolia
NM32	106.76	39.78	Qianlishan Town, Haibowan District, Wuhai City, Inner Mongolia
NM33	106.76	39.71	Wuyi Township, Haibowan District, Wuhai City, Inner Mongolia
NM34	106.78	39.69	Xiahaibowan Town, Haibowan District, Wuhai City, Inner Mongolia
NM35	106.79	39.67	Haibowan District, Wuhai City, Inner Mongolia
NM36	106.78	39.59	Binhe Street, Haibowan District, Wuhai City, Inner Mongolia
NM37	106.76	39.53	Wuda District, Wuhai City, Inner Mongolia
NX1	106.80	39.30	Queergou Village, Huinong District, Shizuishan City, Ningxia
NX2	106.79	39.25	yuanyi Town, Huinong District, Shizuishan, Ningxia
NX3	106.79	39.24	yuanyi Town, Huinong District, Shizuishan, Ningxia
NX4	106.86	39.10	Huinong District, Shizuishan City, Ningxia
NX5	106.87	39.06	Hongyazi Township, Pingluo County, Shizuishan City, Ningxia

NX6	106.78	38.91	Hongyazi Township, Pingluo County, Shizuishan City, Ningxia
NX7	106.72	38.85	Pingluo County, Shizuishan City, Ningxia
NX8	106.64	38.78	Gaoren Township, Pingluo County, Shizuishan City, Ningxia
NX9	106.61	38.72	Gaoren Township, Pingluo County, Shizuishan City, Ningxia
NX10	106.59	38.66	Pingluo County, Shizuishan City, Ningxia
NX11	106.55	38.51	Pingluo County, Shizuishan City, Ningxia
NX12	106.53	38.46	Han Tombs in Xingqing District, Yinchuan City, Ningxia
NX13	106.41	38.36	Lingwu County, Yinchuan City, Ningxia
NX14	106.33	38.28	Hongliu Town, Yongning County, Yinchuan City, Ningxia
NX15	106.25	38.21	Wanghong Town, Yongning County, Yinchuan City, Ningxia
NX16	106.16	38.01	Chen yuantan Town, Wuzhong City, Ningxia
NX17	106.13	37.98	Zaoyuan Township, Wuzhong City, Ningxia
NX18	106.07	37.95	Qinqu Township, Wuzhong City, Ningxia
NX19	106.00	37.89	Qingtongxia Tourist Area in Wuzhong, Ningxia
NX20	105.92	37.83	Guangwu Township, Qingtongxia City, Wuzhong, Ningxia
NX21	105.88	37.62	Miaozitan, Baima Township, Wuzhong City, Ningxia

NX22	105.86	37.61	Xin Qu Shao Cun, Shikong Town, Zhongning County, Ningxia
NX23	105.76	37.56	Tongzhuang Village, Shikong Town, Zhongning County, Ningxia
NX24	105.72	37.54	Shi Kong Zhen Shi Ying Cun, Zhongning County, Ningxia
NX25	105.67	37.53	Zhongning Yellow River Bridge, Zhongning County, Zhongwei City, Ningxia
NX26	105.58	37.50	Yellow River Iron Bridge, Yuding Township, Zhongning County, Zhongwei City, Ningxia
NX27	105.50	37.50	Xuanhe Town, Zhongwei City, Ningxia
NX28	105.43	37.52	Changtan Township, Zhenluo Town, Zhongwei City, Ningxia
NX29	105.35	37.50	Luo Village, Zhenluo Town, Zhongwei City, Ningxia
NX30	105.25	37.49	Dukou Village, Rouyuan Town, Zhongwei City, Ningxia
NX31	105.18	37.48	Binhe Town, Shapotou District, Zhongwei City, Ningxia
NX32	105.12	37.49	Yingshuiqiao Town, Shapotou District, Zhongwei City, Ningxia
NX33	105.06	37.46	Ma Tou Village, Shapotou District, Zhongwei City, Ningxia
NX34	104.99	37.43	Xiaowan, Shapotou District, Zhongwei City, Ningxia
NX35	104.95	37.41	Daliushu Village, Shapotou District, Zhongwei City, Ningxia
NX36	104.93	37.40	Hanhekou, Shapotou District, Zhongwei City, Ningxia
QH1	96.84	35.03	Ma Duo Xiang Zha Jia Cun, Qumalai County, Qinghai Province

QH2	97.92	35.08	Near Zhalinghu Township Government, Maduo County, Qinghai Province
QH3	98.07	35.01	Zhalinghu Township, Maduo County, Qinghai Province
QH4	98.17	34.89	Hydrological Station along the Yellow River, Maduo County, Qinghai Province
QH5	98.35	34.83	Huang He Xiang Re Qu Cun, Maduo County, Qinghai Provinc
QH6	99.66	33.77	Shang Gong Ma Xiang Wang Ri Hu Cun, Gande County, Qinghai Province
QH7	99.73	33.81	Ke Qu Zhen Dang Cheng Cun, Gande County, Qinghai Provinc
QH8	101.18	33.79	Muxihe Township, Maqu County, Gannan, Gansu
QH9	102.25	33.45	Cairima Township, Maqu County, Gannan, Gansu
QH10	102.20	33.84	Manrima Township, Maqu County, Gannan, Gansu
QH11	100.64	34.69	Lajia Town, Maqin County, Qinghai Province
QH12	100.23	35.15	Zhongtie Township, Haixing County, Qinghai Province
QH13	100.46	35.73	Magetang Village, Mangla Township, Guinan County, Qinghai Provinc
QH14	101.49	36.08	Ga Rang Xiang Xi Ji Tan Cun, Guide County, Qinghai Provinc
QH15	101.55	36.12	Erlian Village, Garang Township, Guide County, Qinghai Provinc
QH16	101.94	36.07	Yashiga Town, Hualong County, Qinghai Province
QH17	101.98	36.03	Qun Ke Zhen Xin Cun Yi Cun, Hualong County, Qinghai Provinc

QH18	102.04	35.97	Jianzha County, Qinghai Province
QH19	102.05	35.94	Jianzha County, Qinghai Province (east bank of the Yellow River)
QH20	102.17	35.85	Galeng Xizangan Nationality Township, Xunhua County, Qinghai Provinc
QH21	102.42	35.88	Ji Shi Zhen Yi Ma Mu Cun, Xunhua County, Qinghai Provinc
